# Supplementary material for: The RNA-binding protein Adad1 is necessary for germ cell maintenance and meiosis in zebrafish
Source: PLoS Genet. 2023 Aug 8;19(8):e1010589. doi: 10.1371/journal.pgen.1010589 (PMC10437952; doi:10.1371/journal.pgen.1010589)
Supplement: S1 Table — (PDF) [file pgen.1010589.s001.pdf]

Log2 Fold change of known/likely germ cell-expressed genes in mutants compared to wild types. Genes with differential expression in both mutants are shown in bold.

		Y67X		M392K	
**Regulation**	**Gene name**	**log2 Fold Change**	**Adj.Pval**	**log2 Fold Change**	**Adj.Pval**
	adad2	-0.540788921	0.273955639	-3.272552494	4.19E-06
**Down**	**cdk21**	**-1.136389533**	**0.02926451**	**-2.283816499**	**2.82E-02**
	dazl	-0.845941401	0.083240239	-2.067867757	0.018996408
	ddx4	-0.519925543	0.228314776	-2.267653886	1.79E-04
	dmrt1	-0.057170663	0.898509751	-0.232226754	0.611300819
	dnd1	-1.211297738	0.108023814	-4.256236543	7.39E-10
**Down**	**fkbp6**	**-1.53043257**	**0.005183502**	**-3.797456348**	**8.56E-09**
	hormad1	-0.567949101	0.367910785	-0.773009513	0.504734592
	igf2bp3	0.228730659	0.831318018	-1.826876894	9.03E-04
	mlh1	-0.509506579	0.274646442	-0.406771541	0.348591609
	mlh3	-0.60065428	0.265473561	-0.909194234	0.200168221
	moto (meioc)	-0.502915015	0.321555409	-1.667202938	3.89E-02
**Down**	**nanog**	**-0.851900883**	**0.0333848**	**-5.876920376**	**4.58E-11**
**Up**	**nanos1**	**1.443868276**	**0.032250107**	**0.778065506**	**0.00867982**
	nanos2	-0.129941644	0.885315698	-1.891273476	0.227253548
	nanos3	-1.175949186	0.307025514	-6.145960216	1.38E-09
	piwil1	-0.613315359	0.161723753	-2.710714191	0.000298243
	piwil2	-0.418308801	0.354816732	-2.464766375	0.000247527
	rad21l1	-0.944066301	0.204435606	-2.671782769	1.43E-03
	rad51	-0.644352788	0.12836728	-1.46154254	0.000102198
**Down**	**rad51b**	**-1.234636693**	**1.08E-02**	**-0.79700416**	**0.01814419**
	rec8a	-0.34342045	0.77062949	-2.211187102	0.002198681
	rec8b	-1.121090915	0.178066729	-0.128440531	0.940278319
**Down**	**sall4**	**-1.002308665**	**1.56E-02**	**-3.390435648**	**6.79E-08**
	smc1b	-0.771019266	0.148619356	-1.106449502	0.249313033
	spo11	-1.132101832	0.060017921	-3.097491815	0.000653142
	sycp1	0.722803965	0.406423386	-0.867745504	0.293927194
	sycp2	-0.117321193	0.859870497	-0.475633884	0.755014076
	sycp3	-0.627730014	0.326470135	-0.133518623	0.926655958
	tdrd12	-0.210776278	0.686115375	-3.120134168	0.000154198
	tdrd7b	-2.134558845	0.058763087	-1.248510032	0.383782382
**Down**	**zar1**	**-1.690009196**	**0.032960135**	**-5.592969558**	**4.93E-13**

Log2 fold change of genes listed in table 2-5 are shown. Down-regulated genes in both mutant alleles compared to wild types.

		Y67X		M392K	
**Regulation**	**Gene name**	**log2 Fold Change**	**Adj.Pval**	**log2 Fold Change**	**Adj.Pval**
**Down**	adad1	-4.352617964	3.45E-40	-2.815892698	0.000153256
**Down**	rbp1	-2.95059831	0.000128872	-4.071618415	1.88E-06
**Down**	usp21	-2.682907647	8.14E-05	-3.179468025	0.046991055
**Down**	tdgf1	-2.646116419	3.28E-09	-3.983461671	1.81E-07
**Down**	sumo2a	-2.166748428	0.000610185	-6.506909205	1.89E-15

**Down**	plppr3b	-1.805080024	2.99E-02	-2.441724785	7.85E-03
**Down**	ccna1	-1.730492641	4.72E-02	-6.863267457	1.03E-04
**Down**	zar1	-1.690009196	3.30E-02	-5.592969558	4.93E-13
**Down**	lmx1a	-1.656437252	2.85E-02	-3.605253187	3.80E-04
**Down**	fkbp6	-1.53043257	0.005183502	-3.797456348	8.56E-09
**Down**	hsd17b12a	-1.496134383	4.09E-02	-4.629684258	5.73E-09
**Down**	cpeb1b	-1.39267869	2.97E-02	-6.048428423	1.59E-11
**Down**	rad51ap1	-1.37858447	1.99E-02	-1.442491776	6.46E-03
**Down**	tdrd6	-1.374543896	2.79E-02	-3.536305021	7.73E-07
**Down**	bspry	-1.335878293	1.98E-02	-2.014643004	5.61E-03
**Down**	pdcd2	-1.327735243	1.97E-02	-2.45864841	8.30E-03
**Down**	hsf2bp	-1.325052525	2.24E-02	-2.263558536	1.84E-02
**Down**	sox21a	-1.31616413	4.91E-02	-5.552420208	3.96E-07
**Down**	swsap1	-1.311712033	6.40E-03	-2.021760978	1.46E-03
**Down**	rbm11	-1.306018251	3.35E-02	-2.978403458	1.36E-05
**Down**	abcb5	-1.302270668	1.67E-02	-3.967697206	2.25E-02
**Down**	hsd17b7	-1.292603925	3.62E-04	-1.886196965	1.38E-03
**Down**	mei4	-1.279619159	6.09E-03	-1.533591983	3.83E-02
**Down**	helq	-1.277644489	2.56E-02	-1.49808351	1.33E-02
**Down**	rad51b	-1.234636693	1.08E-02	-0.79700416	1.81E-02
**Down**	mcm8	-1.199754864	2.92E-02	-1.214201381	4.54E-03
**Down**	nudt1	-1.192519465	9.48E-03	-0.925784014	3.41E-02
**Down**	cdk21	-1.136389533	0.02926451	-2.283816499	2.82E-02
**Down**	trip13	-1.124502683	4.33E-02	-1.638433039	7.68E-04
**Down**	fermt1	-1.124093677	4.09E-02	-2.303492476	2.27E-06
**Down**	polr3f	-1.104503046	8.59E-04	-1.682591645	7.86E-05
**Down**	ercc1	-1.055631051	6.12E-03	-0.713147013	1.38E-02
**Down**	tsn	-1.045734753	2.04E-02	-1.455269876	1.30E-04
**Down**	bard1	-1.043919617	4.78E-03	-2.086212586	6.70E-03
**Down**	eif4e1b	-1.034784525	2.55E-02	-4.165822865	4.46E-09
**Down**	eme1	-1.02733536	1.79E-02	-2.16790086	3.88E-07
**Down**	magoh	-1.018133711	9.67E-03	-1.505138772	2.59E-05
**Down**	trdmt1	-1.003500896	3.39E-03	-1.940036719	2.78E-03
**Down**	sall4	-1.002308665	1.56E-02	-3.390435648	6.79E-08
**Down**	tdrd5	-0.946481117	2.55E-02	-3.154663276	2.42E-08
**Down**	nanog	-0.851900883	0.0333848	-5.876920376	4.58E-11
