# Supplementary material for: The RNA-binding protein Adad1 is necessary for germ cell maintenance and meiosis in zebrafish
Source: PLoS Genet. 2023 Aug 8;19(8):e1010589. doi: 10.1371/journal.pgen.1010589 (PMC10437952; doi:10.1371/journal.pgen.1010589)
Supplement: S2 Table — (PDF) [file pgen.1010589.s002.pdf]

S2 Table. Genotyping primers

Gene	Allele	Primer name	Primer sequence
slc34a1a	I423N	KS554	GCTAAAGTGTCACCGGACGTA
slc34a1a	I423N	KS555	TGGAGCCCAGAGTTAGAGGA
slc34a1a	umb10	KNI201	TGATCAGGTCAGACTCAGCAGT
slc34a1a	umb10	KNI202	ACATCCAGACGACACTAAGCTG
adad1	t30103 (M392K)	KS552	GTCAGTATGTCCGCCACTGAT
adad1	t30103 (M392K)	KS553	TTGTACCGTGTCTGGGAAAAG
adad1	sa14397 (Y67X)	KNI203	TTCCCCGTGAGTTGATTGACAGTTA
adad1	sa14397 (Y67X)	KNI204	GCATGCCCTGCAGTGTAATA
