# Supplementary material for: The RNA-binding protein Adad1 is necessary for germ cell maintenance and meiosis in zebrafish
Source: PLoS Genet. 2023 Aug 8;19(8):e1010589. doi: 10.1371/journal.pgen.1010589 (PMC10437952; doi:10.1371/journal.pgen.1010589)
Supplement: S3 Table — (PDF) [file pgen.1010589.s003.pdf]

S3 Table. RT-PCR Primers

Gene	Primer name or reference	Primer sequence
slc34a1a	KNI205	AGTCTGAACCGAGCGTATCC
slc34a1a	KNI206	AACACAGCAGCAGGTACAGC
adad1	KNI207	GTCAGTATGTCCGCCACTGAT
adad1	KNI208	TCTGTGGATTTTCCCTCCAG
ziwi	(1)	CTCAGATGGTGGTGGTGATCT
ziwi	(1)	ACGGTCACACTGTTCCTTCAG
rpl13a	(2)	TCTGGAGGACTGTAAGAGGTATGC
rpl13a	(2)	AGACGCACAATCTTGAGAGCAG
cyp11c1	(3)	GAGCTGATCAGAGCCAACATC
cyp11c1	(3)	GAACTCCAGAACAAGCACAGC

References

1. Siegfried KR, Nüsslein-Volhard C. Germ line control of female sex determination in zebrafish. Dev Biol [Internet]. 2008;324(2):277–87. Available from: http://dx.doi.org/10.1016/j.ydbio.2008.09.025
2. Tang R, Dodd A, Lai D, McNabb WC, Love DR. Validation of zebrafish (Danio rerio) reference genes for quantitative real-time RT-PCR normalization. Acta Biochim Biophys Sin (Shanghai). 2007;39(5):384–90.
3. Wang XG, Orban L. Anti-Müllerian hormone and 11 β-hydroxylase show reciprocal expression to that of aromatase in the transforming gonad of zebrafish males. Dev Dyn an Off Publ Am Assoc Anat. 2007;236(5):1329–38.
