# Supplementary figures and images for: The RNA-binding protein Adad1 is necessary for germ cell maintenance and meiosis in zebrafish
Source: PLoS Genet. 2023 Aug 8;19(8):e1010589. doi: 10.1371/journal.pgen.1010589 (PMC10437952; doi:10.1371/journal.pgen.1010589)

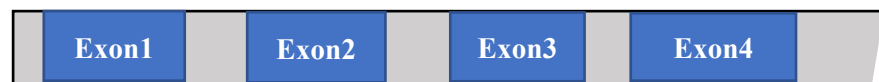

sa14397  
T<A

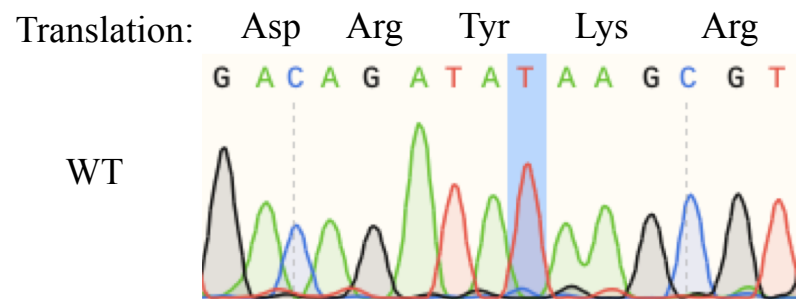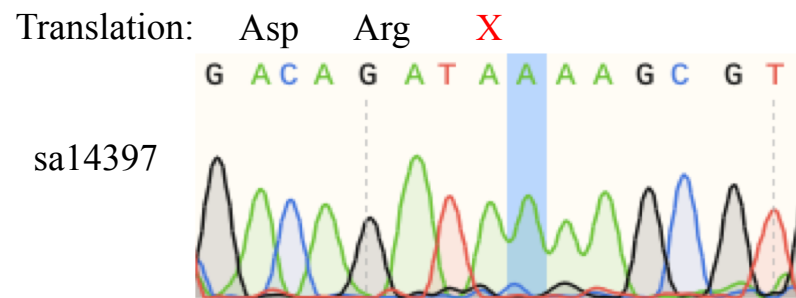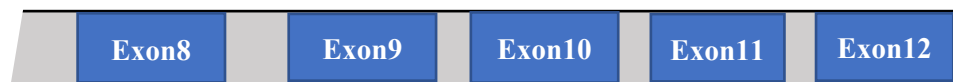

t30103  
T<A

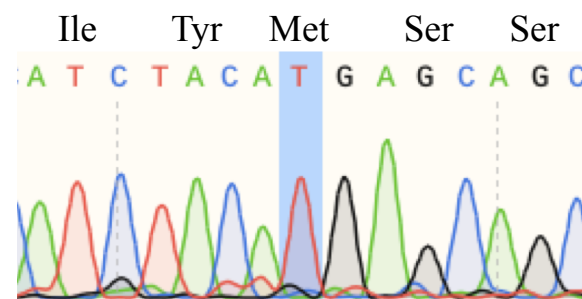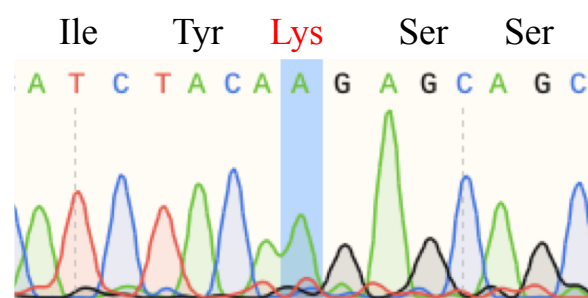

Supplement: S1 Fig — The nonsense mutation (sa14397) is located in exon 3 where a T<A mutation caused a premature stop codon (left panel). The missense mutation (t30103) is in exon 9 where a T<A mutation resulted in a Methionine to Lysine residue change (right panel). (PDF) [file pgen.1010589.s005.pdf]

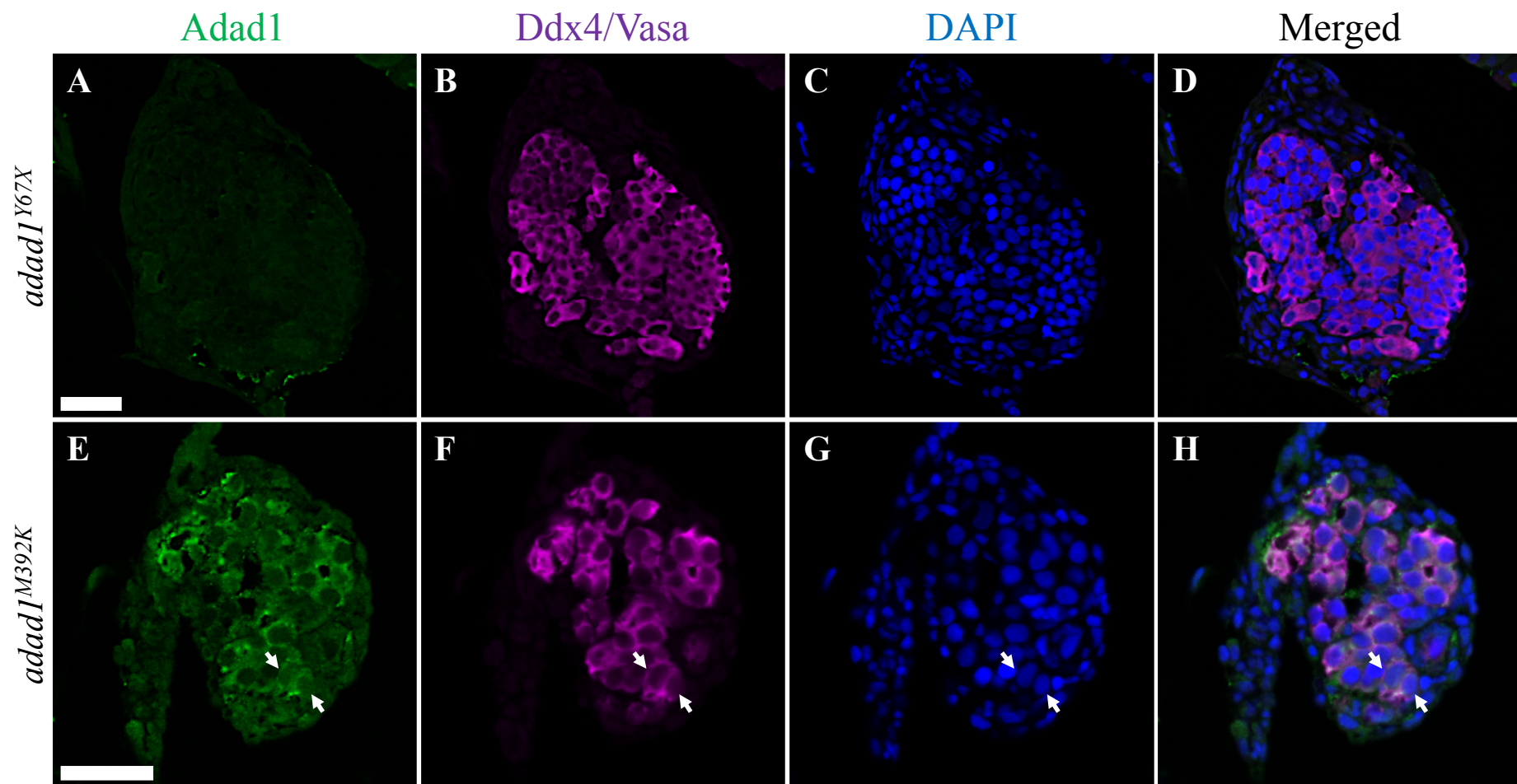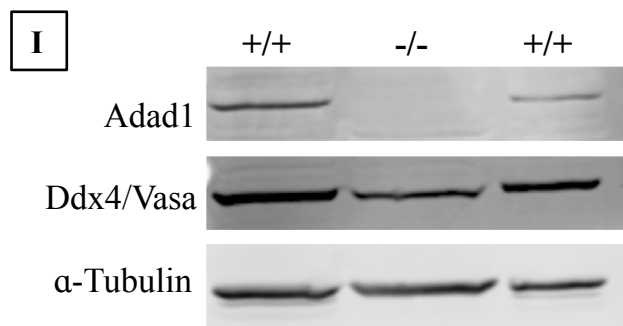

Supplement: S3 Fig — A-D: Immunolabeling of Adad1 antibody on 60 dpf adad1Y67X mutant testis. No protein was detected as expected since the antibody epitope is at the C-terminus of the protein (A). Co-labeling with Ddx4/Vasa showed the presence of germ cells in the mutant testis (B). E-H: Immunolabeling of Adad1 on 45 dpf adad1M393K mutant testis (E) co-labeled with Ddx4/Vasa (F). Arrows point to cells with nuclear and cytoplasmic Adad1 localization. I: Western blot showing that the antibody recognized the 62 kDa size Adad1 protein in wild-type testes extracts but not in testes extracts from adad1Y67X mutants. The wild-type sample was loaded in two lanes: the left lane had twice the volume as the right lane. Ddx4/Vasa was detected in the mutant sample indicating that germ cells were present. Scale bar: 20 μm. (PDF) [file pgen.1010589.s007.pdf]

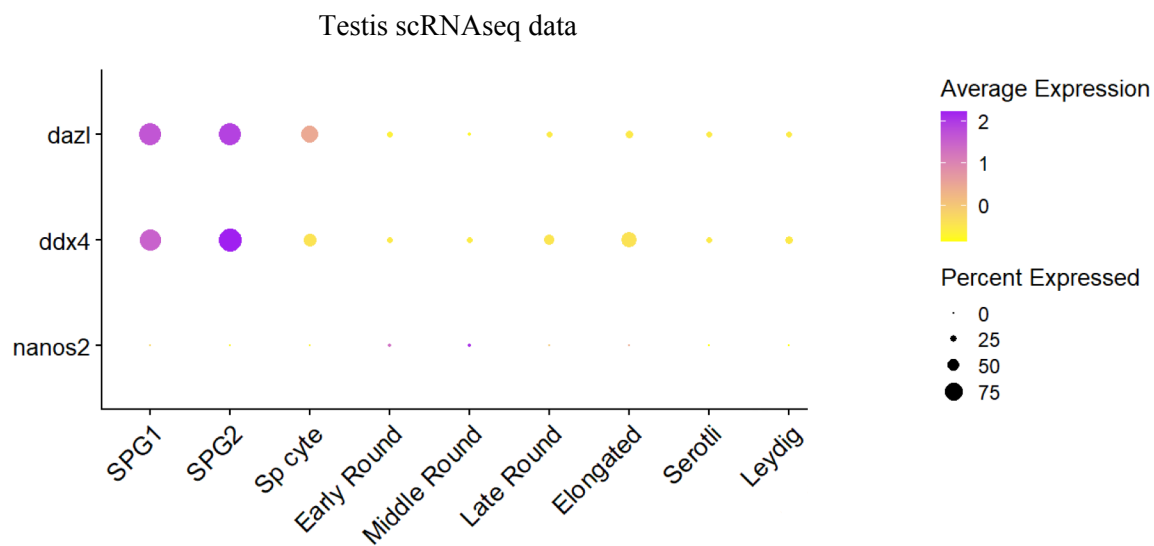

Supplement: S4 Fig — The nanos2 gene was not enriched in spermatogonial cell clusters SPG1 or SPG2 whereas known spermatogonia-expressed genes ddx4 and dazl were enriched in both. (PDF) [file pgen.1010589.s008.pdf]
