# Supplementary material for: The RNA-binding protein Adad1 is necessary for germ cell maintenance and meiosis in zebrafish
Source: PLoS Genet. 2023 Aug 8;19(8):e1010589. doi: 10.1371/journal.pgen.1010589 (PMC10437952; doi:10.1371/journal.pgen.1010589)

```
slc34a1a_WT     ATATCTTCCAGGACAGTGTGATCCTGTCCAACCCGGTGGCGGGGCTGATGGTGGGCATTC
slc34a1a_umb10  ATATCTTCCAGGACAGTGTGATCCTGT-----------------CTTATGGTGGGGATTC
                ***************************                 ** ******** ****
```

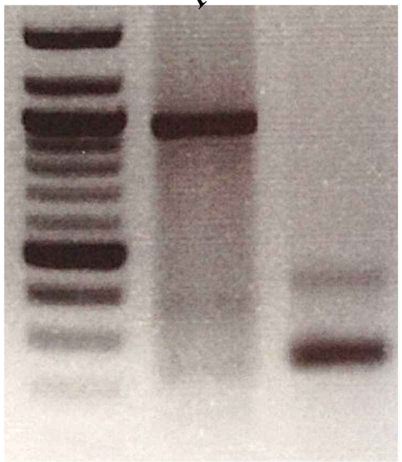

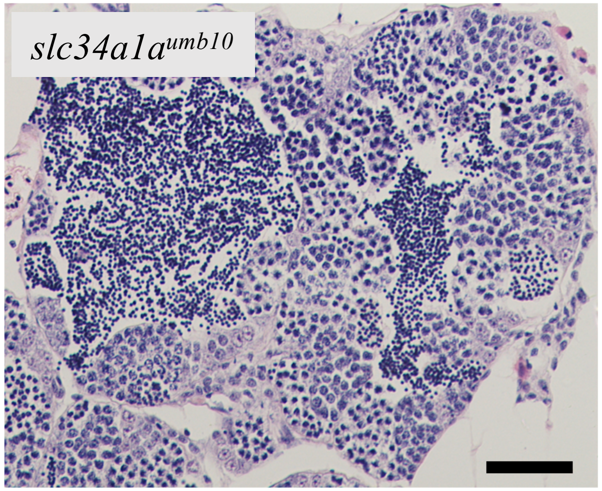

Supplement: S2 Fig — The scl34a1aumb10 mutation is a 17 bp deletion (top panel). The guide-RNA target sequence, including the PAM, is highlighted in yellow. RT-PCR detected slc34a1a expression in adult testes (left panel). Homozygous slc34a1aumb10 mutants exhibited normal testes histology. Scale bar:50 μm. (PDF) [file pgen.1010589.s006.pdf]
