# Supplementary material for: The RNA-binding protein Adad1 is necessary for germ cell maintenance and meiosis in zebrafish
Source: PLoS Genet. 2023 Aug 8;19(8):e1010589. doi: 10.1371/journal.pgen.1010589 (PMC10437952; doi:10.1371/journal.pgen.1010589)

A **Genes from table 2: Pluripotency and stem cell genes downregulated in *adad1* mutants**

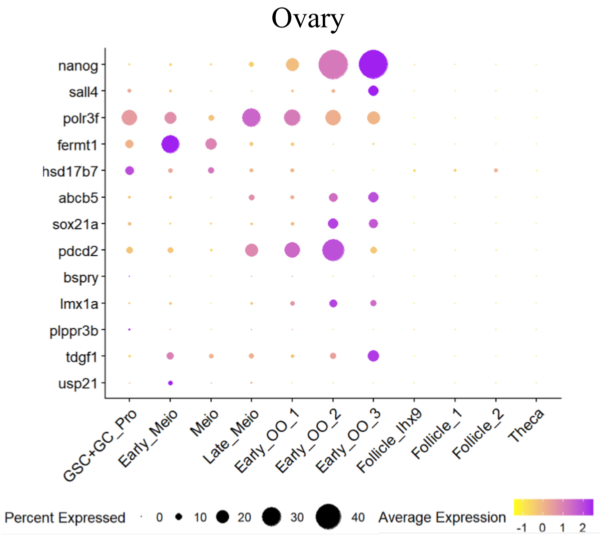

B **Genes from table 5. RNA binding and modification genes downregulated in *adad1* mutants**

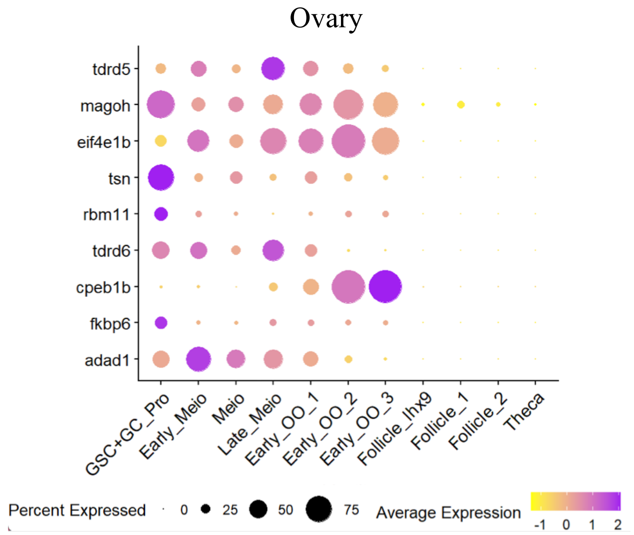

C **Genes from table 3: DNA repair and meiotic recombination genes downregulated in *adad1* mutants**

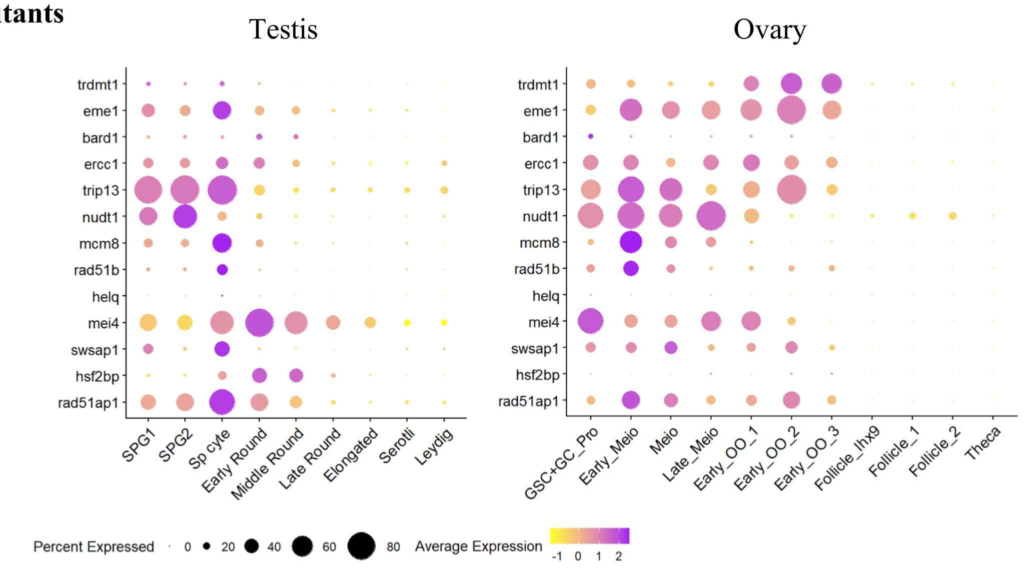

D **Genes from table 4: Meiotic genes downregulated in *adad1* mutants**

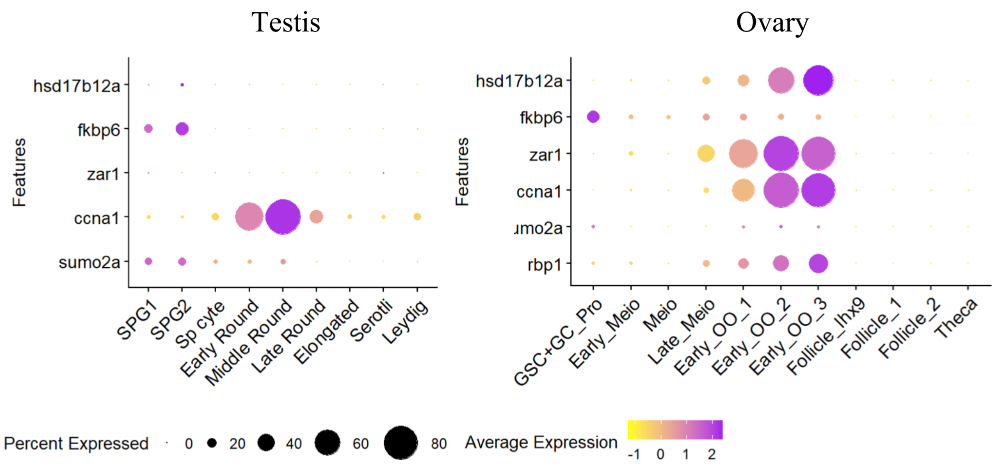

Supplement: S5 Fig — A-D: Dot plot analysis of scRNAseq data from adult testes and 40dpf ovaries. Genes that are listed in Tables 2–4 are shown. (PDF) [file pgen.1010589.s009.pdf]
